# Advances in the Biosynthetic Regulation and Functional Mechanisms of Glycine Betaine for Enhancing Plant Stress Resilience

**DOI:** 10.3390/ijms26167971

**Published:** 2025-08-18

**Authors:** Jiaxu Chen, Jing Zhang, Yihang Liu, Kailu Zhang, Fuyuan Zhu, Yanjie Xie

**Affiliations:** 1 National Key Laboratory for the Development and Utilization of Forest Food Resources, Co-Innovation Center for Sustainable Forestry in Southern China, State Key Laboratory of Tree Genetics and Breeding, Key Laboratory of State Forestry and Grassland Administration on Subtropical Forest Biodiversity Conservation, College of Life Sciences, Nanjing Forestry University, Nanjing 210037, China; 2241800205@njfu.edu.cn (J.C.); jingzhang@njfu.edu.cn (J.Z.); lyh021106@njfu.edu.cn (Y.L.); klzhang@njfu.edu.cn (K.Z.); 2 Laboratory Center of Life Sciences, College of Life Sciences, Nanjing Agricultural University, Nanjing 210095, China

**Keywords:** glycine betaine, plant stress tolerance, biosynthetic regulation, functional mechanisms

## Abstract

Plants are frequently exposed to a range of abiotic stresses, including drought, salinity, extreme temperatures, and heavy metals, that severely impair their growth and productivity. Among the adaptive mechanisms that plants have evolved, the accumulation of glycine betaine (GB), a naturally occurring, zwitterionic, and chemically stable osmoprotectant, has been widely recognized as a key strategy for stress tolerance. In higher plants, GB is primarily synthesized via the two-step oxidation of choline, catalyzed by choline monooxygenase (*CMO*) and betaine aldehyde dehydrogenase (*BADH*). GB contributes to cellular homeostasis by modulating osmotic balance, regulating ion flux, scavenging reactive oxygen species (ROS), enhancing antioxidant defense systems, and stabilizing proteins and membrane structures. Both exogenous application of GB and genetic engineering approaches aimed at enhancing endogenous GB biosynthesis have been shown to significantly improve plant tolerance to a variety of abiotic stresses. In this review, we provide a comprehensive overview of recent advances in the understanding of GB biosynthesis, its regulatory mechanisms, and its multifaceted roles in plant stress responses. We also highlight emerging prospects for leveraging GB-centered strategies to enhance crop resilience in challenging environmental conditions.

## 1. Introduction

Global climate change has markedly increased the frequency and intensity of abiotic stresses, including drought, salinity, and extreme temperatures, which impose continuous challenges on plant systems, severely limiting their growth, development, and overall productivity [1]. These environmental stressors contribute substantially to global agricultural yield losses. In response, plants have evolved a range of adaptive mechanisms, among which the accumulation of compatible solutes, such as amino acids and their derivatives, plays a pivotal role in protecting cellular function under adverse conditions [2].

Glycine betaine (GB), chemically defined as N,N,N-trimethylglycine, is a classic compatible solute [3]. Its zwitterionic structure, comprising a positively charged trimethylammonium group and a negatively charged carboxyl group, confers high solubility and chemical stability. GB can accumulate at substantial concentrations within plant cells without interfering with normal metabolic processes, while significantly enhancing tolerance to osmotic stress, high- and low-temperature stress, and oxidative damage [4,5]. In recent years, GB has attracted increasing attention as a multifunctional protective agent in plant stress physiology. A comprehensive understanding of its biosynthetic pathways, regulatory networks, and physiological roles is essential for developing sustainable strategies to improve crop resilience. Such knowledge could inform the targeted use of GB in agriculture and environmental management, advancing efforts to mitigate the impact of abiotic stresses in a changing climate.

## 2. Biosynthetic Pathways and Regulatory Mechanisms of GB in Plants

### 2.1. Choline Oxidation Pathway

**Figure 1:**
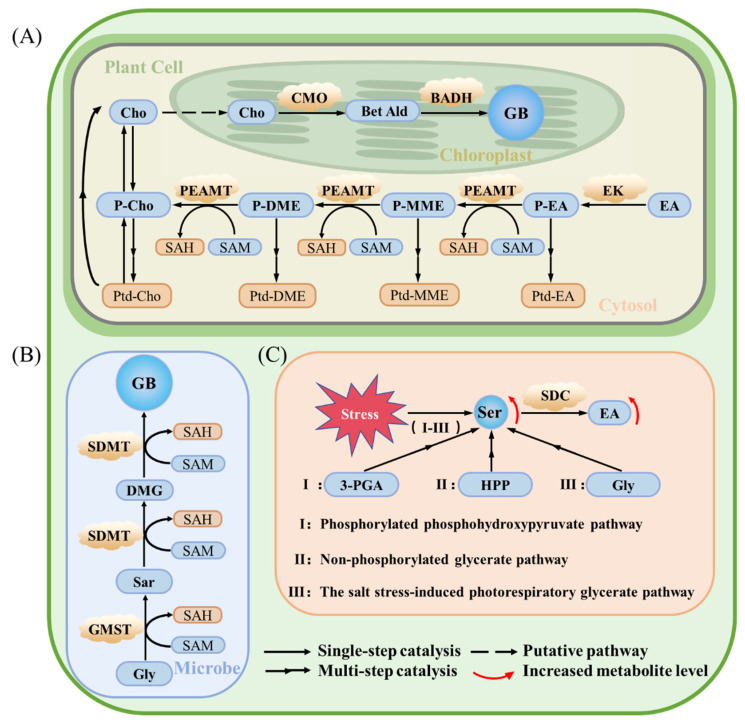
Comparative schematic of the choline oxidation and glycine methylation pathways contributing to GB biosynthesis in plants and microbes. (**A**) The choline oxidation pathway in higher plants. (**B**) The glycine methylation pathway in microbes. (**C**) The serine metabolism pathway under stress. Abbreviations: 3-PGA, 3-phosphoglycerate; *BADH*, betaine aldehyde dehydrogenase; Bet Ald, betaine aldehyde; Cho, choline; *CMO*, choline monooxygenase; DMG, dimethylglycine; EA, ethanolamine; EK, ethanolamine kinase; GB, glycine betaine; Gly, glycine; GSMT, glycine/sarcosine N-methyltransferase; HPP, hydroxypyruvate; P-Cho, phosphocholine; P-DME, phosphodimethylethanolamine; P-EA, phosphoethanolamine; PEAMT, phosphoethanolamine N-methyltransferase; P-MME, phosphomonomethylethanolamine; Ptd-DME, phosphatidyl-dimethylethanolamine; Ptd-Cho, phosphatidylcholine; Ptd-EA, phosphatidylethanolamine; Ptd-MME, phosphatidyl-monomethylethanolamine; SAH, S-adenosylhomocysteine; SAM, S-adenosylmethionine; Sar, sarcosine; SDC, serine decarboxylase; SDMT, sarcosine/dimethylglycine N-methyltransferase; SHMT, serine hydroxymethyltransferase.

The choline oxidation pathway constitutes the primary route for GB biosynthesis in most higher plants (Figure 1A). This pathway proceeds via two sequential enzymatic steps. First, choline is hydroxylated to betaine aldehyde by choline monooxygenase (*CMO*), a stress-inducible, ferredoxin-dependent monooxygenase localized in the chloroplast stroma [6,7]. For example, in salt- and drought-tolerant species like sugar beet (*Beta vulgaris*) and spinach, *CMO* transcript and protein levels increase by 3- to 5-fold during drought and up to 7-fold under high salinity (400 mM NaCl), highlighting its pivotal role in initiating GB biosynthesis in response to abiotic stress [8].

*CMO* requires Fe^2+^, O_2_, and NADPH as cofactors (Figure 1A). In photosynthetic electron transport, ferredoxin (Fd) receives electrons from PSI and donates them to ferredoxin-NADP^+^ reductase (FNR), which reduces NADP^+^ to NADPH, an essential electron donor for *CMO* activity [9,10]. As a result, *CMO* function is tightly coupled to the efficiency of photosynthesis. Indeed, stress-induced damage to the photosynthetic apparatus can impair ferredoxin reduction and NADPH production, thereby hindering GB synthesis [11]. This connection highlights that effective GB biosynthesis requires an intact photosynthetic system.

Following betaine aldehyde formation, the second step of GB biosynthesis involves its irreversible oxidation to GB, catalyzed by betaine aldehyde dehydrogenase (*BADH*), an NAD^+^/NADP^+^-dependent enzyme predominantly localized in the chloroplast stroma [12,13]. Notably, plant *BADH*s generally exhibit higher catalytic activity with NAD^+^ as cofactor [12,14,15], allowing partial functional independence from light-driven reducing power. Overexpression of *BADH* strongly enhances GB accumulation and confers improved abiotic stress tolerance. For instance, transgenic wheat lines overexpressing *HvBADH1* accumulated up to 21.82-fold more GB under salinity stress (150 mM NaCl) [16], while *OsBADH1-*overexpressing rice lines similarly exhibited increased GB content and enhanced salt tolerance [17]. Conversely, *BADH*-deficient mutants show severely impaired GB biosynthesis and heightened stress sensitivity. These findings underscore *BADH* as a critical determinant of GB-mediated stress tolerance in plants.

In most plants, GB biosynthesis proceeds through the choline oxidation pathway. However, in some microorganisms, alternative enzymes mediate the initial oxidation of choline. For example, in certain Gram-negative bacteria (e.g., *Acinetobacter baumannii*), choline is oxidized by choline dehydrogenase (CDH), known as BetA [18]. Other microbes, such as the fungus *Arthrobacter globiformis*, employ choline oxidase (COX) for the same purpose [19]. Nevertheless, *BADH* remains the conserved enzyme responsible for the subsequent conversion of betaine aldehyde to GB in both plant and microbial systems.

Importantly, not all plants possess a fully functional GB biosynthetic pathway (Figure 1B). Genomic analyses of *Oryza sativa* (rice) and *Arabidopsis thaliana* reveal divergence in their *CMO* and *BADH* gene families. Rice contains a *CMO* and at least two *BADH* homologs. The rice *CMO* localizes to the chloroplast and is likely responsible for the initial oxidation of choline [20]. Among its *BADH* homologs, *OsBADH1* is peroxisomal and may oxidize acetaldehyde [21], while *OsBADH2* is implicated in aroma biosynthesis [22]. In *Arabidopsis*, the *CMO* homolog *AtCMO* is weakly expressed, and two *BADH-like* genes, *AtALDH10A8* and *AtALDH10A9*, encode enzymes localized to chloroplasts and peroxisomes, respectively, both capable of oxidizing betaine aldehyde [23,24,25]. Crucially, both *Arabidopsis* and rice accumulate negligible levels of GB under normal or stress conditions due to low *CMO* protein abundance [24]. Overexpression studies have shown that introducing *CMO* alone is insufficient for substantial GB production unless sufficient *BADH* activity is also present [26]. For example, tobacco engineered with expressing *SpCMO* (from spinach) failed to accumulate GB unless co-expressed with a functional *BADH* gene [27]. Therefore, successful metabolic engineering of GB biosynthesis in non-accumulator plants requires coordinated expression of both *CMO* and *BADH* [24]. Indeed, transgenic *Arabidopsis* [28], rapeseed [24], tobacco (*Nicotiana tabacum*) [29], and potato [30] expressing *CMO* and *BADH* together exhibited significantly elevated GB levels and improved stress tolerance.

The transcription of *CMO* and *BADH* is regulated by a complex array of cis-regulatory elements (CREs) responsive to environmental and hormonal signals [31]. Bioinformatic analyses of promoter regions in rice and *Arabidopsis* have identified enrichment of abscisic acid-responsive elements (ABREs), dehydration-responsive elements (DREs), low-temperature responsive elements (LTREs), and antioxidant response elements (AREs), suggesting that both genes are tightly integrated into the convergence of stress-signaling networks [32,33]. Additionally, promoters often contain light-responsive motifs (e.g., G-box, GT1-motif), tissue-specific elements (e.g., ROOTMOTIF), and hormone-associated sites (e.g., TGA-element, CGTCA-motif), reflecting intricate spatial and temporal regulation.

DNA methylation and histone acetylation are key epigenetic mechanisms that regulate chromatin architecture, mediating long-lasting changes in gene accessibility and transcriptional activity [34,35,36,37]. Increasing evidence shows that these modifications, including DNA methylation and diverse histone marks, critically regulate stress-responsive gene expression in plants [38]. Although direct evidence linking GB biosynthetic genes, such as *BADH* and *CMO*, and epigenetic regulation remains elusive, this connection is a plausible and compelling hypothesis, especially under abiotic stress conditions where rapid, reversible gene regulation is vital.

Beyond gene regulation, substrate availability, particularly of choline, is a major control point in GB biosynthesis. In transgenic tobacco overexpressing *SpCMO*, GB accumulation was limited by insufficient endogenous choline levels. Supplementation with 5 mM choline or phosphocholine significantly enhanced both intracellular choline and GB content [27]. Choline supply in plants originates from either de novo synthesis or phospholipid degradation. De novo biosynthesis involves sequential methylation of phosphoethanolamine by phosphoethanolamine N-methyltransferase (PEAMT), producing phosphocholine (PCho), which is then dephosphorylated to yield free choline [39]. In *Beta vulgaris*, phospholipid turnover and the cytidine diphosphate (CDP)-choline pathway constitute the major sources of choline. This pathway involves PEAMT-mediated PCho formation, followed by conversion to CDP-choline via phosphocholine cytidylyltransferase (CCT), and its incorporation into phosphatidylcholine by choline phosphotransferase (CPT). Subsequent hydrolysis of phosphatidylcholine by phospholipases releases free choline for GB synthesis [40]. In contrast, non-accumulator plants like tobacco rely primarily on the PEAMT pathway. Overexpression of PEAMT in tobacco increased phosphocholine levels 5-fold and free choline by 50-fold without affecting membrane lipid content or plant growth [39], underscoring the feasibility of enhancing substrate flux to support engineered GB biosynthesis.

Although GB biosynthesis, particularly via the *CMO* pathway, requires reducing power derived from photosystem I (PSI), GB itself plays a protective role in maintaining photosynthetic efficiency, especially the function of photosystem II (PSII), under stress conditions. This apparent paradox is addressed by several regulatory and physiological features that allow GB synthesis to be sustained even when photosynthesis is partially impaired.

Under abiotic stress (e.g., salinity, drought, cold), PSII is more vulnerable than PSI, which enables limited electron flow through PSI to persist and maintain *CMO* activity. Moreover, GB specifically stabilizes the oxygen-evolving complex of PSII, protects D1 protein from degradation, and promotes its turnover, thereby facilitating PSII repair and recovery [41,42]. This creates a positive feedback loop, in which GB biosynthesis initially relies on PSI-derived electrons, but the accumulated GB helps restore or preserve PSII activity, indirectly supporting sustained photosynthetic function and further GB production.

In addition, the second step of GB synthesis, catalyzed by *BADH*, can utilize NAD^+^, a cofactor not directly linked to photosynthetic electron flow, indicating a partial uncoupling from PSI under stress. Experimental evidence further supports this flexibility: in sugar beet and sorghum, the expression of *CMO* and *BADH* is strongly upregulated under salinity and drought, leading to continued GB accumulation despite reduced carbon fixation and water availability [8,43]. In *Suaeda liaotungensis*, stress-inducible elements in the *BADH* promoter region further enhance its transcription in response to NaCl [44], suggesting that transcriptional regulation compensates for the transient suppression of *CMO* activity during acute stress. These findings together suggest that while GB synthesis depends on photosynthesis for reducing power, the pathway exhibits regulatory plasticity and resilience, allowing for continued GB accumulation that in turn supports and protects the photosynthetic machinery, a finely tuned feedback mechanism crucial for stress adaptation.

### 2.2. Glycine Methylation Pathway

An alternative GB biosynthetic pathway exists in certain halophilic microorganisms such as *Ectothiorhodospira halochloris* and *Actinopolyspora halophila*, where GB is synthesized via the glycine methylation pathway rather than choline oxidation. In this pathway, glycine undergoes three successive S-adenosylmethionine (AdoMet)-dependent methylation reactions, yielding sarcosine, dimethylglycine (DMG), and ultimately GB [45] (Figure 1B). This multi-step pathway was successfully transferred to *Escherichia coli*: heterologous expression of microbial genes encoding glycine betaine methyltransferase (GSMT) and sarcosine/dimethylglycine methyltransferase (SDMT) enabled GB synthesis in *E. coli* even without choline, greatly improving salt tolerance [46,47]. These findings underscore the biotechnological potential of the glycine methylation pathway as an alternative route for GB biosynthesis. Its independence from choline and compatibility with microbial chassis systems make it a promising candidate for synthetic biology applications aimed at improving abiotic stress tolerance in plants and microbes.

No native homologs of these methyltransferases are known in higher plants. However, expressing microbial GSMT and SDMT in plants that cannot synthesize GB represents a novel strategy for enabling them to produce GB. For example, Waditee et al. (2005) engineered *Arabidopsis* by introducing ApGSMT and ApSDMT from the halophilic archaeon Aphanothece halophytica [48]. The transgenic plants accumulated significant GB in roots, stems, leaves, and flowers, levels exceeding those in lines expressing only choline-oxidizing enzymes. Likewise, Ref. [49] introduced *Mpgsmt* and *Mpsdmt* from *Methanohalophilus portucalensis* into *Arabidopsis*, successfully enabling betaine production in plants. These examples demonstrate that the glycine methylation pathway can be harnessed to confer GB biosynthesis in crops lacking the native choline oxidation pathway.

Beyond plants, mammals and certain microorganisms also possess choline oxidation systems functionally analogous to those found in plants. In some Gram-negative bacteria, such as *Acinetobacter baumannii*, choline oxidation is catalyzed by choline dehydrogenase (BetA) [18]. In *E. coli*, choline is converted to GB through the sequential actions of choline dehydrogenase (CDH), encoded by betA, and *BADH*, encoded by betB [50,51]. In contrast, certain eukaryotic microorganisms and fungi, such as *Arthrobacter globiformis*, employ a distinct enzyme, choline oxidase (COX), to catalyze this process [19].

Additionally, GB may also be synthesized through stress-induced pathways (Figure 1C), potentially originating from serine derived via (i) the non-phosphorylated glycerate pathway, (ii) the phosphorylated phosphohydroxypyruvate pathway [52,53], or (iii) the salt stress-induced photorespiratory glycerate pathway [54,55], as proposed by Annunziata [56]. Serine is subsequently converted into Ethanolamine via serine decarboxylase. These pathways link primary metabolism with osmoprotectant biosynthesis under abiotic stress.

### 2.3. Mechanisms of GB Uptake and Transporters in Plants

Efficient GB biosynthesis, whether from endogenous metabolism or exogenous application, depends not only on the availability of choline but also on its intracellular transport, particularly into the chloroplast where GB synthesis occurs. Cytosolic choline, whether synthesized de novo or absorbed from the environment, must be effectively delivered to the sites of GB biosynthesis.

Plants possess multiple transport systems for choline. These include a high-affinity choline transporter (CHT1), choline transporter-like (CTL) proteins of intermediate affinity, and low-affinity organic cation transporters (OCTs) [57]. The first plant choline transporter identified was *AtCTL1* from *Arabidopsis thaliana*, which mediates choline uptake. Intriguingly, *AtCTL1* also influences auxin distribution by promoting the trafficking of auxin efflux carriers, linking choline transport to developmental processes. The *Arabidopsis* CTL1 ortholog in roots, known as Choline Transporter1 (CHER1), may similarly facilitate choline transport to maintain root growth [58]. Therefore, GB production depends not only on the amount of cytosolic choline but also on how efficiently it is imported into chloroplasts [59,60]. Supporting this, Yamada et al. (2011) used a yeast mutant deficient in choline uptake to show that sugar beet Proline/GB transporters (Pro/BeTs) have a higher affinity for choline than for GB [61]. These findings highlight a crucial layer of regulation in GB metabolism at the level of membrane transport, linking substrate availability with organellar import and stress-induced demand.

In bacteria, the osmoprotective choline transporter BetT provides a model. *E. coli* uses BetT for choline uptake, enabling subsequent GB synthesis. Yang et al. (2024) solved the cryo-EM structure of BetT, revealing how it binds choline [62]. Such structural insights could guide engineering of plant transport systems to improve GB accumulation. To date, no plant protein has been identified that specifically transports GB across membranes, and the H^+^-coupled transporters that might move GB remain to be discovered [42,63].

At the whole-plant level, experiments show that GB applied to one part can move to others. Park et al. (2006) demonstrated that applying GB to a mature tomato leaf resulted in rapid transport of GB via the phloem to meristematic tissues, such as flower buds and shoot apices [64]. Similarly, GB-accumulating transgenic *Arabidopsis* [65] and tomato [66] plants actively translocated foliar GB through the phloem to growing sinks [64,66]. This phloem mobility suggests that exogenous GB can distribute throughout the plant, reaching target tissues to confer stress protection where it is most needed.

## 3. Functional Roles of GB in Plant Stress Tolerance Mechanisms

**Figure 2:**
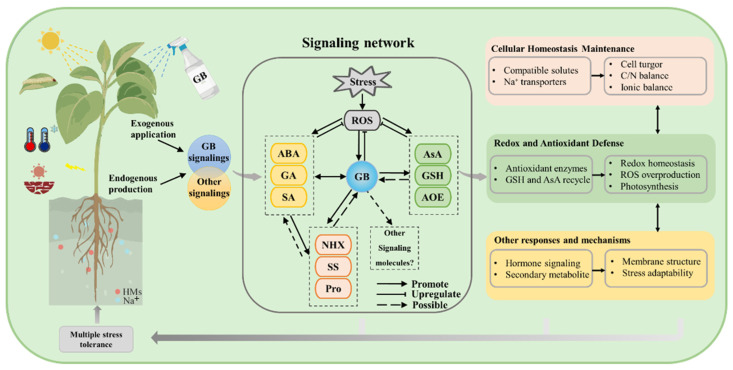
Mechanisms of GB action in regulating plant responses to multiple stresses (excessive light, pest infestations, temperature extremes, soil-related constraints). GB enhances plant tolerance to multiple stresses including drought, salinity, high temperatures, and insect herbivory. Abbreviation: ABA, abscisic acid; AOE, antioxidant enzymes; AsA, ascorbic acid; GA, gibberellic acid; GSH, glutathione; HKT, Na^+^/H^+^ antiporter; HMs, heavy metals; Pro, proline; ROS, reactive oxygen species; SA, salicylic acid; SS, soluble sugar.

GB enhances plant tolerance to diverse abiotic stresses through a suite of coordinated physiological and molecular mechanisms (Figure 2). Under stress conditions, GB modulates key phytohormonal signaling pathways, including abscisic acid (ABA), gibberellins (GA), and salicylic acid (SA), thereby regulating stress-responsive gene expression and adaptive responses. It contributes to osmotic and ionic homeostasis by facilitating Na^+^ exclusion and promoting the accumulation of compatible solutes such as proline and soluble sugars. GB also strengthens antioxidant defenses by activating enzymes like superoxide dismutase (SOD) and catalase (CAT) and by enhancing the ascorbate-glutathione (AsA-GSH) cycle for efficient reactive oxygen species (ROS) detoxification. Furthermore, GB helps preserve photosynthetic efficiency and membrane stability through transcriptional regulation and hormone-mediated crosstalk. Notably, GB accumulation may also deter herbivory by interfering with insect feeding behavior, suggesting a broader role in enhancing plant resilience under combined abiotic and biotic stresses (Figure 2).

### 3.1. Maintenance of Cellular Homeostasis

#### 3.1.1. Regulation of Osmotic Adjustment

Osmotic adjustment is one of the earliest and most critical cellular responses to abiotic stress [67]. Under conditions such as salinity and drought, the external water potential declines, leading to cellular dehydration and turgor loss. GB, due to its high solubility and metabolic inertness, accumulates in the cytosol without interfering with normal biochemical processes [68,69]. Its accumulation enables cells to retain water and maintain turgor pressure, thereby sustaining metabolic activity and physiological function under osmotic stress.

Moreover, GB can stimulate the accumulation of other compatible solutes such as proline and soluble sugars. Under salt stress, exogenous GB has been shown to upregulate the expression of pyrroline-5-carboxylate synthetase (P5CS), a key gene in proline biosynthesis, and enhance sucrose phosphate synthase (SPS) activity, resulting in elevated levels of proline and sugars [70]. The combined effect of GB, proline, and sugars synergistically reduces cellular osmotic potential and helps maintain turgor under drought and salinity stress. Thus, GB functions not only as a primary osmolyte but also as a regulatory molecule that coordinates the synthesis of additional osmoprotectants to support water balance in stressed plants.

#### 3.1.2. Regulation of Ion Homeostasis

Maintaining ion homeostasis is essential for cellular function, particularly under salt stress, where excessive accumulation of Na^+^ and Cl^−^ can impair enzymatic activity and disrupt membrane potentials. GB contributes to ionic balance by enhancing the selective uptake of beneficial ions such as K^+^ and promoting the exclusion or vacuolar compartmentalization of toxic ions [71]. This regulatory effect helps preserve cellular ionic equilibrium and supports metabolic stability under saline conditions.

At the molecular level, GB treatment upregulates the expression of potassium transporter genes. For instance, members of the K^+^ uptake permease (KUP) family (e.g., Potassium transporter 21-like) are induced by GB, enhancing both high- and low-affinity K^+^ uptake [72]. This helps maintain an optimal cytosolic K^+^ concentration and a favorable K^+^/Na^+^ ratio, which is essential for enzyme function and cell turgor. Under salt stress, GB-treated plants tend to accumulate more K^+^ in roots and restrict Na^+^ transport to shoots [73]. Additionally, GB can activate H^+^/K^+^ co-transport systems and stimulate Na^+^ efflux pumps, further reducing Na^+^ toxicity. Through these actions, GB helps maintain stable ion gradients and preserves metabolic and signaling functions during salt stress.

#### 3.1.3. Regulation of Redox Homeostasis

Redox homeostasis entails a dynamic balance between ROS production and antioxidant defense mechanisms [74]. Abiotic stresses frequently disrupt this balance, leading to excessive ROS accumulation and oxidative damage to lipids, proteins, and DNA. GB contributes to redox regulation both by directly scavenging ROS and by enhancing endogenous antioxidant systems. For example, exogenous GB application in rice seedlings significantly increased glutathione reductase (GR) and ascorbate peroxidase (APX) activities by 30% and 25%, respectively, thereby reinforcing the ascorbate-glutathione (AsA-GSH) cycle and limiting H_2_O_2_ accumulation [75]. In pepper, GB treatment upregulated genes encoding superoxide dismutase (CaSOD), peroxidase (CaPOD), catalase (CaCAT), CaGR1, and dehydroascorbate reductase (CaDHAR) [76]. Through these mechanisms, GB mitigates oxidative stress and helps maintain redox homeostasis under adverse environmental conditions.

#### 3.1.4. Stabilization of Membrane Structures

Membrane stability is essential for maintaining cellular compartmentalization, transport, and signal transduction [77]. Under abiotic stress, lipid peroxidation and phase transitions can disrupt membrane architecture and impair functionality. GB helps preserve membrane stability by interacting with lipid bilayers, enhancing their fluidity and thermal resilience. In chloroplasts, GB maintains the fluidity of thylakoid membranes and prevents deleterious phase changes under heat or salinity stress. This stabilization effect protects PSII complexes, thereby supporting sustained electron transport and maintaining photosynthetic efficiency under adverse conditions [78].

GB similarly stabilizes mitochondrial membranes: it maintains crista architecture and prevents the collapse of membrane potential and disassembly of respiratory complexes [75,79]. These actions ensure efficient ATP synthesis under stress. At the molecular level, GB is thought to stabilize membrane-associated proteins as well, thereby preserving ion gradients, enzyme activities, and signaling processes essential for survival under adverse conditions. By reinforcing membrane stability across organelles, GB helps safeguard cellular structures during stress.

### 3.2. Regulation of Metabolic Networks

Beyond maintaining cellular homeostasis, GB modulates broader metabolic networks to support plant performance under stress. Under high temperatures or salt stress, GB mitigates photoinhibition and enhances photosynthetic efficiency by stabilizing photosynthetic enzymes and membrane systems. Specifically, GB specifically interacts with thylakoid lipids to stabilize PSII reaction centers, thereby preserving the structural integrity and functionality of the photosynthetic apparatus [80]. It also regulates chloroplast membrane fluidity [42], thereby reducing high light- or heat-induced inhibition of electron transport and limiting the degradation of key photosynthetic components [41]. Collectively, these effects contribute to improved energy conversion efficiency and sustained photosynthetic activity under abiotic stress.

Maintenance of carbon–nitrogen (C/N) metabolic balance is another crucial aspect of stress adaptation [81]. Under normal conditions, carbon assimilation (photosynthesis) provides sugars and energy, while nitrogen assimilation (nitrate uptake and assimilation) generates amino acids and proteins. Abiotic stresses often disrupt this balance by suppressing photosynthesis and nitrogen metabolism, leading to ROS buildup and metabolic imbalance [82]. GB helps reestablish C/N balance by enhancing key carbon and nitrogen metabolic enzymes. For example, under moderate drought conditions, foliar application of 25, 50, and 100 mM GB increased the soluble sugar content in sweet potato leaf tissues by 1.98-, 2.21-, and 2.37-fold, respectively, compared to the control [70]. The accumulated sugars (from carbon metabolism) and proline (from nitrogen metabolism) act as osmolytes and energy stores [83].

In addition to facilitating metabolic adjustment, GB modulates the synthesis of secondary metabolites that enhance plant stress defenses. GB has been shown to induce the biosynthesis of flavonoids and lignin, two key compounds involved in abiotic and biotic stress tolerance. Flavonoids function as potent antioxidants that scavenge ROS, while lignin reinforces cell walls, improving mechanical strength and pathogen resistance [84]. Interestingly, genes involved in GB biosynthesis share structural or regulatory features with those governing phenylpropanoid metabolism, including lignin and other phenolic compound pathways [85,86]. This suggests a possible metabolic or transcriptional linkage, whereby GB signaling may coordinate broader defensive metabolite networks. Such cross-pathway regulation further enhances antioxidant capacity and structural resilience, contributing to overall stress adaptation.

### 3.3. Enhancement of Plant Tolerance to Abiotic Stress by GB

#### 3.3.1. Drought Stress

Drought is a major stress that disrupts plant water balance, reduces water-use efficiency (WUE), and compromises photosynthesis, ultimately limiting growth. It notably affects chloroplasts, where water deficit induces an over-reduction in the photosynthetic electron transport chain, resulting in ROS accumulation. This accumulation damages cellular integrity and alters the chloroplast proteome [87]. Specifically, drought stress can harm chloroplast membranes, increase ROS levels, and inhibit chlorophyll biosynthesis along with the expression of chlorophyll-binding proteins, thereby compromising PSII function [88,89].

Exogenous GB application can ameliorate drought-induced damage through multiple physiological layers. GB-treated plants often show optimized stomatal behavior, enhanced photosynthesis, stronger antioxidant defenses, and improved energy metabolism, all contributing to reduced water loss under drought. For example, in cotton (*Gossypium hirsutum*), Hamani et al. (2021) found that foliar GB improved stomatal conductance, gas exchange, and chlorophyll fluorescence under drought conditions [90].

At the cellular level, GB helps maintain membrane potential and water status in both the cytosol and the apoplast. It acts as an antioxidant, scavenging excess drought-induced ROS and preventing membrane damage [91]. Additionally, GB supports the photosynthetic machinery: it promotes turnover of the PSII D1 protein, alleviating photodamage and sustaining chlorophyll content and photosynthetic efficiency [92].

At the molecular level, GB influences gene expression related to cell wall and energy metabolism. Bai et al. (2022) reported that GB upregulates *Cellulose Synthase A* (*CesA*) to enhance cellulose biosynthesis, which may improve water retention. GB also modulated the expression of enzymes in energy pathways, diacylglycerol acyltransferase (DGAT) and glycerol-3-phosphate dehydrogenase (GPDH), and affected endogenous abscisic acid (ABA) levels via genes such as *phospholipase D* (*PLD*) and *glutamyl-tRNA synthetase* (*GluRS*) [93]. These changes suggest that GB coordinates osmotic signaling with metabolic adjustments under drought. In another example, applying 20 mM GB to Indian mustard (*Brassica juncea*) markedly improved drought resilience [94]. GB-treated mustard plants exhibited higher growth, biomass, leaf water content, nutrient uptake, and photosynthetic performance compared to controls. They also accumulated more osmolytes, exhibited stronger antioxidant enzyme activity, maintained better ion balance, and had higher cell viability under water stress. This comprehensive response confirms that GB mitigates drought stress via coordinated physiological and molecular changes.

#### 3.3.2. Salt Stress

Salt stress poses multiple challenges to plants. Excess external Na^+^ disturbs cellular ion homeostasis by competing with K^+^ uptake and directly inhibits enzymes and protein synthesis. These disruptions lead to reduced growth, lower biomass, and yield losses. To cope, plants minimize water loss, enhance water uptake, sequester Na^+^ in vacuoles, or actively extrude Na^+^ to alleviate toxicity [95].

GB aids salt stress tolerance primarily by acting as an osmoprotectant that maintains ionic and osmotic balance [73]. Exogenous GB treatments have been shown to improve biomass and growth under salinity by increasing WUE, raising the K^+^/Na^+^ ratio, enhancing photosynthesis, bolstering ROS scavenging, and inducing stress-related genes [96,97]. For example, in maize, GB-treated plants upregulated the plasma membrane H^+^-ATPase, which helps extrude Na^+^ and thus mitigates ion toxicity [97]. In tobacco, GB reduced salt-induced oxidative damage by lowering protein carbonylation levels [98]. In Stevia (*Stevia rebaudiana*), GB application under salt stress improved growth by modulating nitrogen metabolism, polyamine oxidase activity, and antioxidant defenses while maintaining K^+^/Na^+^ homeostasis [99]. Intriguingly, GB also increased the expression of enzymes in the steviol glycoside biosynthesis pathway, leading to higher levels of rebaudioside A (a major sweet compound) under salinity. This suggests GB might induce protective secondary metabolites in response to salt stress. Together, these studies show that GB contributes to salt tolerance through multiple layers: it preserves osmotic balance across membranes and simultaneously upregulates antioxidant systems to prevent oxidative injury. These combined effects sustain growth and photosynthesis in saline environments.

Recent advances have expanded GB applications via nanotechnology. Ganjavi et al. (2021) created glycine betaine-loaded graphene oxide (GO-GB) nanoparticles and demonstrated that they promoted growth and mitigated salt stress in sweet basil [100]. Similarly, Hanif and Zia (2023) found that combined application of GB and zinc oxide nanoparticles (ZnO NPs) in plants significantly reduced Na^+^ accumulation and increased K^+^ levels. This combination improved ionic homeostasis and lessened salt damage, suggesting that nano-formulations can enhance GB’s efficacy in stress mitigation [101].

#### 3.3.3. Temperature Stress

Cold stress induces ice crystal formation in plant tissues, disrupting cellular functions. Foliar application of GB acts as a cryoprotectant, enhancing cold tolerance by upregulating antioxidant defenses, protecting photosynthesis, and modulating hormone and cold-responsive pathways [102]. For example, in tomato seedlings, GB treatment increased *catalase1* (*CAT1*) expression and activity, reduced ROS levels, preserved PSII function, and improved photosynthetic efficiency under chilling conditions [64]. Regarding hormonal balance, GB elevates ABA while suppressing GA, thereby enhancing seed cold tolerance and germination [103]. Notably, maize studies reveal a feedback loop where endogenous ABA promotes GB accumulation by activating *BADH* [104].

GB also activates cold-responsive transcription factors: it induces *SlCBF* and *ICE1* in tomato, which regulate downstream genes for cold adaptation [102]. Under cold storage of fruits, GB boosts antioxidant defenses. In Chinese pear (*Pyrus ussuriensis* ‘Nanguo’), GB application elevated activities and transcripts of APX, CAT, and SOD, thereby reducing lipid peroxidation caused by chilling and improving fruit quality [105]. It has also been shown to modulate the balance of ABA and GA during seed dormancy, helping seeds overcome early cold stress [103]. Thus, GB’s main role in cold stress appears to be the upregulation of antioxidant systems, coupled with hormonal adjustments, which collectively protect cells from chilling injury.

Heat stress (high temperature) likewise harms plants by denaturing proteins, damaging membranes, and inhibiting photosynthesis [106,107]. GB contributes to thermotolerance through several strategies. It can induce heat shock proteins (HSPs), especially HSP70, which function as molecular chaperones during heat stress [108]. In transgenic tomato, overexpression of *CODA* (a choline oxidase gene from *Arthrobacter globiformis*) led to higher GB levels and greater heat resistance than overexpressing spinach *BADH* alone [109]. These *CODA* plants maintained higher antioxidant enzyme activities, exhibited lower ROS accumulation, and preserved PSII function under heat. They also sustained higher CO_2_ assimilation and PSII efficiency compared to controls. In these plants, H_2_O_2_ may act as a signaling molecule to activate heat stress genes, further enhancing thermotolerance [109]. Foliar GB application in various crops has similarly been shown to boost CAT and SOD activities under heat, reinforcing antioxidant defenses.

In summary, GB provides comprehensive protection against temperature extremes. Under heat, it helps maintain osmotic balance, stimulates HSP accumulation, and activates antioxidant systems, collectively safeguarding cellular homeostasis. Under cold, it primarily fortifies antioxidant defenses and stress signaling pathways to prevent chilling damage. Besides temperature extremes, GB has beneficial effects against other stresses such as heavy metal toxicity. Exogenous GB has been reported to alleviate heavy metal stress (e.g., chromium, cadmium) by improving growth, photosynthesis, and antioxidant enzyme activities, while reducing metal accumulation and oxidative stress in plants [110,111]. These findings suggest that GB’s protective mechanisms extend to a broad range of abiotic challenges.

### 3.4. Enhancing Plant Resistance to Biotic Stress

GB also contributes to biotic stress resistance by interacting with salicylic acid (SA) signaling, a key regulator of systemic acquired resistance (SAR) and pathogenesis-related (PR) protein expression. GB can enhance SA signal transduction, upregulates SA biosynthetic genes such as *ICS1*, and activates the SA regulator *NPR1*, thereby promoting *PR* gene expression and broad-spectrum pathogen resistance [112]. Additionally, GB helps maintain SA pathway homeostasis, preventing excessive defense activation that could hinder growth. Through these synergistic effects, GB-treated plants can more efficiently resist various pathogens, including bacteria and viruses. Additionally, GB has been demonstrated nematocidal activity. Khanna et al. (2019) reported that GB can disrupt nematode cell membranes and metabolism, providing a novel approach to managing nematode-related plant diseases [113,114].

Moreover, GB influences the production of defensive compounds. In mango and tobacco, GB-mediated defense responses involved the transcription factor *MiWRKY53*, which enhanced chalcone isomerase (CHI) activity, a key step in flavonoid biosynthesis. Increased CHI activity correlated with reduced disease symptoms and better chlorophyll retention in infected tissues [115]. In a groundbreaking study, foliar application of GB together with chitosan in cucumber plants greatly reduced symptoms and viral loads of cucumber mosaic virus (CMV) [112]. These results highlight GB’s potential as an inducer of disease resistance and a modulator of plant defense metabolism.

## 4. Strategies for Applying GB to Enhance Plant Stress Resilience

Enhancing GB levels in plants has proven to be an effective strategy for improving plant stress tolerance. Two main approaches are commonly employed: exogenous application and genetic engineering of GB biosynthetic pathways (Figure 2). Both methods increase GB accumulation, activate stress-responsive pathways, and enhance resilience to a range of abiotic stresses.

### 4.1. Exogenous Application of GB

The exogenous application of GB, through foliar spray, seed treatment, or soil drenching, is a practical strategy to enhance plant stress tolerance. Among these, foliar application is especially effective, as GB is efficiently absorbed through leaves, leading to a rapid increase in internal concentration (Table 1). For example, treating peach (*Prunus persica*) with GB induced changes in cellular energy status, raised internal levels of GB, γ-aminobutyric acid (GABA), and proline, and protected membranes, collectively enhancing cold tolerance [116]. In another case, spraying GB on licorice (*Glycyrrhiza uralensis*) seedlings under salt stress stimulated osmolyte accumulation, elevated antioxidant enzyme activities, promoted salt excretion, and significantly increased Na^+^ efflux, thereby improving salt tolerance [117]. Similarly, applying 30 mM GB to Chinese cabbage leaves improved freezing tolerance by reducing membrane damage and ROS accumulation without harming plant health [118].

Exogenous GB acts through multiple regulatory mechanisms. In maize, Bai et al. (2022) showed that GB alleviated drought stress by modulating endogenous ABA levels and stomatal conductance via stress-responsive gene expression [93]. In *Arabidopsis*, Zhang et al. (2022) identified the gene *PCST1* as mediating osmotic stress responses by regulating proline and GB biosynthesis, as well as *SOS*, *NCED*, and *CIPK* genes [146]. In tomato, GB was found to mitigate cold-induced inhibition of seed germination through several signaling pathways [103]. GB application has also improved postharvest fruit quality: it enhanced flavor and cold tolerance in stored peaches [147] and extended the storability and shelf life of Citrus reticulata ‘Huangguogan’ [129].

Despite these benefits, exogenous GB has limitations. Its uptake, distribution, and metabolic fate in plants can vary with species, developmental stage, and environmental conditions, leading to inconsistent stress mitigation. Large-scale application of GB can also be costly and may pose ecological risks, such as unintended effects on soil microbiota and surrounding ecosystems. Careful optimization of dosage, timing, and delivery methods is therefore necessary for practical use.

### 4.2. Genetic Engineering

Genetic engineering provides an alternative strategy to enhance GB accumulation by introducing key biosynthetic enzymes. Overexpression of GB biosynthetic genes has been shown to improve stress tolerance across various species. For example, transgenic soybean lines expressing the *Atriplex hortensis BADH* gene (*AhBADH*) exhibited significantly increased salt tolerance in transgenic lines [148]. Similarly, tobacco plants overexpressing spinach *BADH* demonstrated enhanced resistance to cadmium stress [149].

Interestingly, enzymes in the GB pathway can also impact crop quality traits. In rice, the *BADH2* gene is closely linked to fragrance: mutations that reduce *BADH2* activity increase the aroma compound 2-acetyl-1-pyrroline (2AP) [22,150]. Recent studies have mapped expression and protein QTLs for *BADH2*, revealing its major role in 2AP variation [151]. Novel *BADH2* alleles are being used to breed fragrant rice (Hui et al., 2022). In peanut (*Arachis hypogaea*), CRISPR/Cas9 knockout of *AhBADH1* and *AhBADH2* produced lines with dramatically higher 2AP content and enhanced aroma [152]. These examples show that the manipulation of GB biosynthesis genes can have broader metabolic consequences, affecting secondary traits like flavor.

However, genetic modifications targeting aroma-related genes may also impact plant responses to abiotic stress. For example, under increasing salinity, both the transcript levels of *BADH1* and the *BADH1/BADH2* transcript ratio were significantly elevated in the leaf tissues of both aromatic and non-aromatic rice cultivars. In contrast, *BADH2* transcript levels displayed no consistent pattern in response to salt stress. These observations suggest that *BADH1*, rather than *BADH2*, plays a functional role in mediating salt stress responses in rice [153]. While targeted knockout of the *OsBADH2* gene induces aroma formation, phenotypic responses to salinity vary depending on the genetic background. For instance, CRISPR/Cas9-induced mutants of two non-aromatic rice varieties, Huaidao#5 and Jiahua#1, both developed aromas but exhibited opposite responses to salt stress: enhanced tolerance in Huaidao#5 and reduced tolerance in Jiahua#1 [154]. Physiological and transcriptional analyses indicated differential changes in osmolyte levels and stress-responsive and antioxidant enzymatic gene expression, suggesting that interactions between genetic background and *OsBADH2* disruption play a critical role in determining stress response outcomes. Therefore, further molecular investigations are essential to elucidate the regulatory mechanisms linking *OsBADH* gene function with both aroma biosynthesis and abiotic stress tolerance in rice.

Despite the engineering of GB pathways showing promise, challenges remain. Transgene expression must be stable and robust; factors like genomic integration site, promoter choice, and epigenetics can affect performance. Overexpressing GB biosynthetic enzymes may also disrupt metabolic homeostasis, potentially impairing growth. Therefore, future efforts should focus on understanding the regulatory networks governing GB metabolism and on finding effective tissue-specific or stress-inducible promoters. Optimizing transformation protocols and coordinating GB gene expression with the plant’s overall metabolism will be essential. Only by balancing enhanced stress tolerance with normal development can these approaches achieve practical agricultural benefits.

## 5. Plant GB in Agriculture: Mechanisms, Challenges, and Future Perspectives

As a natural osmoprotectant, GB holds substantial promise for improving crop stress resistance. Whether through endogenous synthesis or exogenous application, GB significantly enhances plant tolerance to a broad spectrum of abiotic and biotic stresses. Elucidating its molecular mechanisms of action is thus a key focus in plant science and crop improvement.

GB is predominantly synthesized via the choline oxidation pathway in plants or via the glycine methylation pathway in certain microbes. The capacity for GB accumulation varies widely among species and is influenced by environmental conditions and developmental stage. Mechanistically, GB maintains cellular osmotic homeostasis and stabilizes membranes and proteins, mitigating water loss and cellular damage under drought, salinity, or temperature extremes. Its antioxidant function is especially noteworthy: GB both directly neutralizes ROS and upregulates enzymes like SOD and CAT, thereby alleviating oxidative cytotoxicity. Additionally, GB modulates carbon-nitrogen metabolism by promoting osmolyte synthesis (e.g., proline, sugars) and sustaining energy supply under stress. Extensive studies have also shown that GB participates in stress signaling. It influences hormone signaling pathways (e.g., ABA and SA) and activates stress-responsive transcriptional networks under drought, salt, and cold. These signaling roles further integrate GB into the plant’s defense responses.

In terms of applications, exogenous GB supply is a simple and effective way to improve plant performance under stress. However, its efficacy depends on uptake and transport efficiency, which vary with plant species, tissue type, and application method. Consequently, stress protection by GB can be inconsistent. Transgenic strategies, in contrast, have achieved promising results: introducing *CMO* and *BADH* genes has enhanced stress tolerance in many crops. Intriguingly, GB biosynthesis genes can also affect quality traits like grain aroma in rice. Yet genetic approaches still face hurdles, such as unstable transgene expression and possible metabolic imbalances. Notably, the mechanisms of GB uptake and transport across plant membranes (e.g., potential H^+^-coupled GB transporters) remain poorly understood, representing a bottleneck for both basic research and practical deployment.

Future research should focus on unraveling the molecular networks regulating GB biosynthesis, signaling, and transport, particularly its crosstalk with plant hormones, transcription factors, and redox regulators. Integrating advanced approaches such as gene editing, synthetic biology, metabolic engineering, and nano-based delivery systems will facilitate precise manipulation of GB accumulation and activity. For example, the development of eco-friendly nano-formulations may enhance the efficiency of GB delivery under field conditions. Despite existing challenges in regulation, application efficiency, and field translation, GB remains a promising metabolic tool for enhancing stress adaptation. Its potential to support stress-resilient crop breeding and promote sustainable agriculture is especially valuable in the context of global climate change and agroecosystem stability [155].

## Figures and Tables

**Table 1:** Summary of the application of glycine betaine on various plant species under abiotic stress.

Stress Type	Species	Exogenous GB Concentration	Resistance Effect	Reference
Drought stress	Chickpea (*Cicer arietinum* L.)	0.17 mM	Increased antioxidant enzyme activity.	[119]
Drought stress	Flax (*Linum usitatissimum*)	50–100 mM	Enhancement on induction of protein, carbohydrate, and ROS scavenging systems.	[91]
Drought stress	Maize (*Zea mays* L.)	0.5–10 mM	Improves water retention and reduces osmotic potential, mitigating drought-induced water loss.	[120]
Drought stress	Indian pennywort (*Centella asiatica*)	25–50 mM	Overall physiological, morphological, and secondary metabolite traits were enhanced.	[121]
Drought stress	Peach (*potted Prunus persica* L.)	0.85–4 mM	Decreased leaf plasma membrane permeability, H_2_O_2_, Pro, and soluble sugar contents, increased leaf ASA-POD activity and soluble protein content.	[122]
Drought stress	Sweet potato (*Ipomoea batatas*)	50–100 mM	Increased sugar content and photosynthetic ability, controlled cellular osmotic potential, and maintained storage root yield.	[70]
Drought stress	Tobacco (*Nicotiana tabacum*)	20 mM	Improved plant growth, osmotic adjustment, photosynthesis, and antioxidant enzyme activities.	[56]
Drought stress	Wheat (*Triticum aestivum*)	98.2 mM	Decreased contents of H_2_O_2_, O^2−^ and MDA, increased antioxidant enzymes, proline, and soluble sugar contents.	[123]
Salt stress	Cowpeas (*Vigna unguiculata*)	5–10 mM	Increased soluble sugar contents and antioxidant enzymes.	[124]
Salt stress	Chinese licorice (*Glycyrrhiza uralensis* Fisch.)	10–40 mM	Enhances antioxidant defense, osmoregulation, and salt excretion capacity.	[117]
Salt stress	Maize (*Zea mays* L.)	0.1 mM	Improved photosynthesis and antioxidant activity.	[97]
Salt stress	Perennial ryegrass (*Lolium perenne*)	20–50 mM	Increased fresh weight and relative water content; reduced electrolyte leakage and malondialdehyde content.	[125]
Salt stress	*Suaeda salsa* (L.)	10–50 mM	Increased soluble sugars and elevated activity of Na^+^, K^+^-ATPase (enhancing osmotic stability).	[126]
Cold stress	Alfalfa (*Medicago sativa*)	200 mM	Decreased ion leakage from shoot tissues.	[127]
Cold stress	Cabbage (*Brassica oleracea* L.)	30 mM	Freezing tolerance is enhanced by reducing membrane leakage, MDA, and ROS without affecting leaf growth.	[118]
Cold stress	Cristalina Cactus Pear (*OPUNTIA ficus-indica* (L.) Mill.)	5 mM	Improved fruit quality through enhanced size, composition, and nutritional content.	[128]
Cold stress	‘Huangguogan’(*Citrus reticulata* Blanco)	10–20 mM	Antioxidant system activation reduces ROS and lipid peroxidation.	[129]
Cold stress	Maize (*Zea mays* L.)	2.5 mM	Prevented chlorosis; reduced lipid peroxidation of membrane.	[130]
Cold stress	Peach (*Prunus persica* Batsch.)	10 mM	Activating arginine/GABA metabolism and inhibiting polyamine degradation.	[116]
Cold stress	Pears (*Pyrus communis* L.)	10 mM	Decreased membrane lipid peroxidation, maintained membrane integrity, increased activities and expression of APX, CAT, SOD, and Pro content.	[105]
Cold stress	Tomato (*Lycopersicon esculentum*)	10 mM	Minimizes cold-induced seed damage by modulating oxidants, metabolites, and hormones to promote germination.	[103]
Heat stress	Barley (*Hordeum vulgare*)	10 mM	Enhances PSII stability by increasing antenna connectivity, protecting the oxygen-evolving complex.	[131]
Heat stress	Mrigold (*Tagetes erecta*)	0.5–1 mM	Improves heat tolerance by protecting the photosynthetic apparatus, increasing stomatal conductance, and enhancing ROS scavenging.	[132]
Heat stress	Pingyi Tiancha (*Malus hupehensis* (Pamp.) Rehder)	10 mM	Improved water status and enhanced antioxidant enzyme activity may underlie GB-induced enhancement of photosynthesis under stress.	[133]
Heat stress	Rice (*Oryza sativa* L.)	10 mM	Boosting antioxidant enzymes, lowering MDA and ROS, and improving osmoregulation to ease heat stress.	[134]
Heat stress	Sugarcane (*Saccharum* sp.)	20 mM	Increasing K^+^ and Ca^2+^ levels, supporting tissue differentiation and dry weight accumulation.	[135]
Heat stress	Tomato (*Lycopersicon esculentum*)	0.1–5 mM	Enhanced expression of heat-shock genes and accumulation of HSPs.	[136]
Heat stress	Wheat (*Triticum aestivum*)	100 mM	Maintenance of higher chlorophyll content, PSII photochemical activity, and net photosynthetic rate.	[137]
Heavy metal stress (Cr)	Cauliflower (*Brassica oleracea* L.)	1 mM	Increased dry biomass and improved antioxidative enzyme activities.	[138]
Heavy metal stress (Cr)	Mung bean (*Vigna radiata*)	50–100 mM	Improved plant growth; alleviated chromium stress.	[139]
Heavy metal stress (Cr)	Sorghum (*Sorghum bicolor* L.)	50–100 mM	Increases antioxidant enzyme activity due to a decrease in chromium uptake or reduction in EL.	[128,140]
Heavy metal stress (Cr)	Wheat (*Triticum aestivum*)	100 mM	Improved growth, chlorophyll contents, and biomass and protein levels under chromium stress.	[141]
Heavy metal stress (Cd)	Cotton (*Gossypium hirsutum* Linn.)	1 mM	Cd toxicity is mitigated by enhanced antioxidant enzyme activity.	[142]
Heavy metal stress (Cd)	Tobacco (*Nicotiana tabacum*)	0.5 mM	Reduced MDA content, induced stomatal closure, improved leaf/root ultrastructure, increased the chl content, Fv/Fm, SOD, POD, CAT, and APX activities.	[143]
Heavy metal stress (Al)	Cucumber (*Cucumis sativus* L.)	100 mM	Accumulating in chloroplasts, GB mitigates Al stress by protecting the photosynthetic apparatus, enhancing electron transport, gas exchange, and CO_2_ fixation.	[144]
Heavy metal stress (Pb)	Pakchoi (*Brassica campestris* L.)	0.5–2 mM	Increased dry biomass, mineral nutrient, and pigment contents, antioxidative enzyme activities, and reduced Pb contents.	[145]
